# The *Salmonella* Genomic Island 1 Is Specifically Mobilized *In Trans* by the IncA/C Multidrug Resistance Plasmid Family

**DOI:** 10.1371/journal.pone.0015302

**Published:** 2010-12-20

**Authors:** Gregory Douard, Karine Praud, Axel Cloeckaert, Benoît Doublet

**Affiliations:** INRA, UR1282, Infectiologie Animale et Santé Publique, Nouzilly, France; New England Biolabs, Inc, United States of America

## Abstract

**Background:**

The *Salmonella* genomic island 1 (SGI1) is a *Salmonella enterica*-derived integrative mobilizable element (IME) containing various complex multiple resistance integrons identified in several *S. enterica* serovars and in *Proteus mirabilis*. Previous studies have shown that SGI1 transfers horizontally by *in trans* mobilization in the presence of the IncA/C conjugative helper plasmid pR55.

**Methodology/Principal Findings:**

Here, we report the ability of different prevalent multidrug resistance (MDR) plasmids including extended-spectrum β-lactamase (ESBL) gene-carrying plasmids to mobilize the multidrug resistance genomic island SGI1. Through conjugation experiments, none of the 24 conjugative plasmids tested of the IncFI, FII, HI2, I1, L/M, N, P incompatibility groups were able to mobilize SGI1 at a detectable level (transfer frequency <10^−9^). In our collection, ESBL gene-carrying plasmids were mainly from the IncHI2 and I1 groups and thus were unable to mobilize SGI1. However, the horizontal transfer of SGI1 was shown to be specifically mediated by conjugative helper plasmids of the broad-host-range IncA/C incompatibility group. Several conjugative IncA/C MDR plasmids as well as the sequenced IncA/C reference plasmid pRA1 of 143,963 bp were shown to mobilize *in trans* SGI1 from a *S. enterica* donor to the *Escherichia coli* recipient strain. Depending on the IncA/C plasmid used, the conjugative transfer of SGI1 occurred at frequencies ranging from 10^−3^ to 10^−6^ transconjugants per donor. Of particular concern, some large IncA/C MDR plasmids carrying the extended-spectrum cephalosporinase *bla*_CMY-2_ gene were shown to mobilize *in trans* SGI1.

**Conclusions/Significance:**

The ability of the IncA/C MDR plasmid family to mobilize SGI1 could contribute to its spread by horizontal transfer among enteric pathogens. Moreover, the increasing prevalence of IncA/C plasmids in MDR *S. enterica* isolates worldwide has potential implications for the epidemic success of the antibiotic resistance genomic island SGI1 and its close derivatives.

## Introduction

Acquisition of foreign DNA by horizontal gene transfer (HGT) is a crucial mechanism that allows bacteria to acquire new traits, and it represents a key driving force in bacterial evolution. HGT is widely recognized as the mechanism responsible for the widespread distribution of antibiotic resistance genes. Among HGT mechanisms, those specified by conjugative plasmids seem to be the most sophisticated and implicated in the diffusion of antibiotic resistance genes [1]. Bacterial conjugation appears to be a conserved molecular process by which transfer of DNA from a donor to a recipient cell occurs during a close cell-cell contact. In Gram-negative bacteria, conjugation requires the elaboration of a type IV secretion system, called the transferosome and the formation of a nucleoprotein complex involved in processing the DNA and delivering it to the transferosome, called the relaxosome [2]. Although conjugation is a conserved mechanism, conjugative systems (transferosome and relaxosome) have shown some differences depending on the incompatibility (Inc) group of plasmids [2]. Incompatibility is a manifestation of the relatedness of plasmids that share common replication controls. Incompatibility was defined as the inability of two related plasmids to be propagated stably in the same bacterial strain [3], [4].

Localization of antimicrobial resistance genes on transferable genetic elements such as broad-host range plasmids, integrative conjugative elements facilitates the HGT of these genes among bacteria and provides a rapid mean of spread [5]. Certain replicon types (correlated with Inc groups) are associated with multidrug resistance (MDR) from bacteria implicated in disease outbreaks or found in food-producing animals [6]. In this context, IncA/C MDR plasmids are widely distributed among foodborne pathogens such as *Salmonella* [7]. IncA/C replicon types are associated with plasmids carrying and disseminating extended-spectrum β-lactamase (ESBL) genes in animals and humans [6]. During the past decade, specific attention has been focused on IncA/C MDR plasmids that encode the AmpC β-lactamase CMY-2 (*bla*_CMY-2_ gene) especially among *Salmonella enterica* and *Escherichia coli* [5], [6], [8], [9].

On the other hand, the 43-kb *Salmonella* genomic island 1 (SGI1) is the first genomic island conferring a MDR phenotype identified in *S. enterica* [10]. SGI1 is an integrative mobilizable element (IME) which contains a complex class 1 integron, named In104, located at the 3′ end of the island [11]. The In104 complex integron confers the common penta-resistance profile to ampicillin (Amp), chloramphenicol (Chl), streptomycin (Str), sulphonamides (Sul) and tetracycline (Tet) of the epidemic MDR *S. enterica* serovar Typhimurium defined phage type DT104 (*S*. Typhimurium DT104) strains [10], [11]. Since the identification of SGI1 in *S.* Typhimurium DT104, variant SGI1 MDR complex integrons have been described in a wide variety of *S. enterica* serovars and more recently also in *Proteus mirabilis* [11]–[14]. The identification of SGI1 in *P. mirabilis* clinical isolates is of great concern as the spread of the SGI1 (or variants) MDR phenotype could have significant implications in other pathogenic bacteria.

In all cases and until now, in field or clinical *S. enterica* and *P. mirabilis* strains, SGI1 is found integrated into the bacterial chromosome within the last 18 bp of the *trmE* gene (also named *thdF*) [10], [11]. In 2005, we reported that SGI1 could be conjugally transferred from *S. enterica* donor strains to non-SGI1 *S. enterica* and *E. coli* recipient strains where it integrated into the recipient chromosome in a site-specific manner [15]. Briefly, after excision of SGI1 from the *Salmonella* chromosome, the conjugative mobilization in *trans* by the conjugative helper IncA/C plasmid pR55 occurs between donor and recipient strains. In the recipient cell, the circular form of SGI1 integrates in a site-specific manner at the 3′ end of the chromosomal *trmE* gene [15]. SGI1 appeared to be a non-self-transmissible but mobilizable element and was thus classified within the group of site-specific integrative mobilizable elements (IMEs) that are related to integrative conjugative elements (ICEs) [15].

In the present study, we report the conjugative *in trans* mobilization assays of SGI1 by several plasmids of different incompatibility groups. We have tested epidemic successful plasmids carrying Extended Spectrum Cephalosporins (ESC) resistance genes encoding CTX-Ms, TEM-52 ESBLs, and CMY-2 AmpC β-lactamase [16]. According to the results of our plasmid collection tested, we found that only the broad-host-range conjugative IncA/C plasmids were able to mobilize *in trans* SGI1. Conjugative IncHI2 and IncI1 plasmids carrying ESBL genes (*bla*_CTX-M_ or *bla*_TEM_) were unable to mobilize SGI1. Although certain IncA/C MDR *bla*_CMY-2_ plasmids seemed to have lost their self-transferable capacity, some conjugative IncA/C MDR *bla*_CMY-2_ plasmids permitted the conjugative mobilization of SGI1 [5], [7], [8]. Our findings suggest that a close relationship, probably at a molecular conjugative process level, may exist between the IncA/C MDR plasmid family and the MDR genomic island SGI1.

## Results and Discussion

The identification of SGI1 in the chromosome of several *S. enterica* serovars, i.e. Agona, Cerro, Derby, Dusseldorf, Emek, Haifa, Infantis, Kedougou, Kentucky, Kiambu, Kingston, Meleagridis, Newport, Paratyphi B, Tallahassee, Typhimurium, Virchow, and also in *P. mirabilis* suggested that SGI1 might be horizontally mobilized by conjugative elements concomitantly borne by field strains [11]–[15]. Mobilization experiments were undertaken to determine whether SGI1 was mobilizable *in trans* by different conjugative plasmids previously described as spreading among MDR *Salmonella* strains and/or other pathogenic Enterobacteriaceae [16]. We firstly introduced by conjugation a set of 17 conjugative ESBL plasmids (CTX-M-1, -2, -9 and TEM-52) of IncHI2 and IncI1 groups in the SGI1-carrying *S.* Agona strain 959SA97 previously described to conjugally transfer SGI1 in the presence of the conjugative IncA/C helper plasmid pR55 (Table 1 and data not shown) [15]. These ESBL IncHI2 and IncI1 plasmids are representative of the ESBL plasmid families that spread among several *S. enterica* serovars in animal and human isolates representing a major concern [17], [18]. In all mobilization assays between ESBL plasmids/SGI1-carrying *S.* Agona strain 959SA97 and the recipient *E. coli* K-12 strain BM14, no SGI1 transconjugants could be obtained in 3 repeated attempts (Table S1 in the supplemental material). In all mating experiments, control of conjugative transfer of the introduced helper plasmid was positive (data not shown). Two TEM-52 IncI1 conjugative plasmids p777SA01 and p04-3486 were initially isolated from *S.* Agona and *S.* Typhimurium field strains that also harboured SGI1 or SGI1-A variant, respectively (Table 1) [17]. Mobilization of SGI1 was also tested from these field strains and resulted also negatively (Table S1). These results indicated that SGI1 is not mobilized by our set of ESBL plasmids of IncHI2 and IncI1 groups from *S. enterica* to *E. coli*. These data suggested that IncHI2 and IncI1 plasmids are not able to mobilize SGI1. Moreover, these results indicated that the presence of any conjugative plasmid, which would provide all conjugal transfer functions for successful conjugation, is not the only sufficient prerequisite for mobilization of SGI1. However, our ESBL plasmid collection is mainly from *S. enterica* sources and does not contain plasmids of other Inc groups than HI2 and I1 that are also described to harbour various ESBL genes (*bla*_TEM_, *bla*_SHV_, *bla*_CTX-M_) in bacterial isolates from other sources such as *E. coli* from humans [19].

**Table 1:** Bacterial strains, and plasmids used in this study.

Strains and plasmid	Relevant genotype, resistance profile^a^, original host or characteristics	Reference or source
***S. enterica*** ** SGI1 donors**		
Agona 959SA97	SGI1^+^; AmpChlStrSulTet	[10]
Agona 959SA97ΔS009::*kan*	SGI1ΔS009::*kan* ^+^; AmpChlKanStrSulTet	[10]
Agona 47SA97	SGI1-C^+^; StrSul	[30]
Agona 47SA97Δ*xis*::*kan*	SGI1-CΔ*xis*::*kan* ^+^; KanStrSul	[30]
Agona 777SA01	SGI1-A^+^; IncI1^+^ (TEM-52); AmpChlStrSulTetTmp-3GC	[17]
Albany 7205.00	SGI1-F^+^; AmpChlSulTetTmp	[31]
Typhimurium 04-3486	SGI1^+^; IncI1^+^ (TEM-52); AmpChlStrSulTet-3GC	[17]
***E. coli*** ** recipient**		
BM14	K-12 J53 derivative; F- *pro met azi*; Az^R^	Institut Pasteur, France
**Plasmids**		
IncA/C pRA1	Tra^+^; SulTet; *A. hydrophila*	[32]
IncA/C pIP40A	Tra^+^; AmpKanSul; *P. aeruginosa*	[33]
IncA/C pR55	Tra^+^; AmpChlGenKanSul; *K. pneumoniae*	[24]
IncA/C pR16a	Tra^+^; AmpKanSul; *P. stuartii*	[21]
IncA/C p13688	Tra^+^; AmpChlGenStrSulTetTmp-3GC; CMY-2; *E. coli*	[9]
IncA/C p13956	Tra^+^; AmpChlStrSulTetTmp-3GC; CMY-2; *E. coli*	[9]
IncA/C pAM04528	Tra^−^; AmpChlStrSulTet-3GC; CMY-2; *S.* Newport	[8]
IncA/C pN418	Tra^−^; AmpChlGenKanStrSulTet-3GC; CMY-2; *S.* Heidelberg	[7]
IncFI pOX38	Tra^+^; Kan; F factor derivative; *E. coli*	Institut Pasteur, France
IncFII R1-16	Tra^+^; Kan; R1 derivative; *E. coli*	[20]
IncHI2 pCEB6542	Tra^+^; SulTet-3GC; CTX-M-1; *S.* Llandoff	[34]
IncHI2 p1639-SA-00	Tra^+^; AmpSulTetTmp-3GC; CTX-M-2; *S.* Virchow	[18]
IncHI2 p142-SA-01	Tra^+^; AmpSulTetTmp-3GC; CTX-M-2; *S.* Virchow	[fernandez2007]
IncHI2 p3464b	Tra^+^; AmpStrSulTetTmp-3GC; CTX-M-9; *S.* Virchow	[35]
IncI1 p777-SA-01	Tra^+^; Amp-3CG; TEM-52; *S.* Agona	[17]
IncI1 p04-3486	Tra^+^; Amp-3CG; TEM-52; *S.* Typhimurium	[17]
IncI1 R112	Tra^+^; Kan; *S.* Panama	[21]
IncL/M R69	Tra^+^; AmpKanTet; *S.* Paratyphi B	[21]
IncN RPC3	Tra^+^; KanStr; *E. coli*	[21]
IncP RP4	Tra^+^; AmpKanTet; *P. aeruginosa*	[33]
IncW RSa	Tra^+^; ChlKanStrSul; *Shigella flexneri*	[22]

a abbreviations: Amp, ampicillin; Az, sodium azide; Chl, chloramphenicol; Gen, gentamicin; Kan, kanamycin; Str, streptomycin; Sul, sulphonamides; Tet, tetracyclines; Tmp, trimethoprim; 3GC, third generation cephalosporin.

In spite of these negative results, we decided to test reference conjugative plasmids of main different incompatibility groups previously described for plasmid typing [20]–[22]. Conjugative plasmids pRA1 (IncA/C), pOX38 (IncFI), R1-16 (IncFII), R112 (IncI1), R69 (IncL/M), RPC3 (IncN), RP4 (IncP), and Rsa (IncW) were introduced by conjugation into SGI1-carrying *S.* Agona strain 959SA97 or SGI1-C-carrying *S.* Agona strain 47SA97 depending on antibiotic selection used to introduce the plasmid and thereafter for the transfer of SGI1 (Table 1). The presence of each reference plasmid in *S.* Agona SGI1 donor strains was confirmed by PCR based-replicon typing [3]. Among all the reference plasmids tested, only the IncA/C reference plasmid pRA1 initially isolated from *Aeromonas hydrophila* in 1971 was able to mobilize variant SGI1-C from *S.* Agona donor strain 47SA97 in mating experiments with the *E. coli* recipient strain BM14 (Table 2). *E. coli* SGI1-C transconjugants showed the antibiotic resistance profile conferred by SGI1-C (StrSul) and the additional resistance to sodium azide. The presence of SGI1 or variants of it was confirmed by several PCRs indicating that the entire SGI1 was presented in *E. coli* transconjugants and integrated into the chromosome (data not shown). The kanamycin resistance conferred by plasmid pRA1 was not transferred to the *E. coli* recipient. The absence of plasmid pRA1 in *E. coli* transconjugants was also confirmed by the specific IncA/C replicon PCR (data not shown) [3]. These results indicated that the IncA/C reference plasmid pRA1 was not transferred into the transconjugants tested and confirmed that SGI1 was mobilized *in trans* by pRA1. Using the IncA/C reference plasmid pRA1 as helper plasmid, *E. coli* SGI1-C transconjugants were found at a frequency of around 10^−3^ (Table 2). All other conjugative reference plasmids of IncFI, IncFII, IncI1, IncL/M, IncN, IncP, and IncW were unable to mobilize SGI1 or variant SGI1-C in repeated mating experiments (Table S1). In all mating experiments, conjugative transfer control of the different conjugative helper plasmids was positive (data not shown). These results suggested that the *in trans* mobilization of SGI1 may be correlated with the presence of a helper conjugative plasmid of the IncA/C family in the *Salmonella* SGI1 donor strain.

**Table 2:** SGI1 mobilization by IncA/C plasmids.

*S. enterica* donor strain		SGI1 variant	Conjugative plasmid	SGI1 transfer frequency^a^	
**Field strain**					
	Agona 959SA97	SGI1	−	<10^−9^	
	Agona 47SA97	SGI1-C	−	<10^−9^	
	Albany 7205.00	SGI1-F	−	<10^−9^	
**Derivative of field strain**					
	Agona 959SA97ΔS009::*kan*	SGI1ΔS009::*kan*	–	<10^−9^	
	Agona 47SA97Δ*xis*::*kan*	SGI1-CΔ*xis*::*kan*	–	<10^−9^	
**Transconjugant strain**					
	Agona 47SA97	SGI1-C	IncA/C pRA1	9.8 10^−3^	
	Agona 47SA97	SGI1-C	IncA/C pIP40a	1.9 10^−2^	
	Agona 959SA97	SGI1	IncA/C pR55	1.9 10^−3^	
	Agona 47SA97	SGI1-C	IncA/C pR55	3.4 10^−3^	
	Albany 7205.00	SGI1-F	IncA/C pR55	7.2 10^−5^	
	Agona 959SA97	SGI1	IncA/C pR16a	4.4 10^−3^	
	Agona 47SA97	SGI1-C	IncA/C pR16a	8.9 10^−3^	
	Agona 47SA97Δ*xis*::*kan*	SGI1-CΔ*xis*::*kan*	IncA/C pR55	3.1 10^−7^	
	Agona 959SA97ΔS009::*kan*	SGI1ΔS009::*kan*	IncA/C p13688 (CMY-2)	6.7 10^−5^	
	Agona 959SA97ΔS009::*kan*	SGI1ΔS009::*kan*	IncA/C p13956 (CMY-2)	2.2 10^−5^	
	Agona 47SA97Δ*xis*::*kan*	SGI1-CΔ*xis*::*kan*	IncA/C p13688 (CMY-2)	5.2 10^−6^	
	Agona 47SA97Δ*xis*::*kan*	SGI1-CΔ*xis*::*kan*	IncA/C p13956 (CMY-2)	2.2 10^−6^	
	Albany 7205.00	SGI1-F	IncA/C pAM04528 (CMY-2)	<10^−9^	
	Albany 7205.00	SGI1-F	IncA/C pN418 (CMY-2)	<10^−9^	

a the frequency of transfer was calculated by dividing the number of SGI1 transconjugants by the number of SGI1 donor cells. Transfer frequencies correspond to the result of one experiment which has been repeated two times and showing the same results.

To assess whether the IncA/C plasmid family is specifically implicated in the mobilization of SGI1, and whether different IncA/C plasmids may mobilize *in trans* SGI1, we introduced by conjugation different MDR IncA/C plasmids into SGI1-carrying *S.* Agona strain 959SA97 or SGI1-C-carrying *S.* Agona strain 47SA97 or SGI1-F-carrying *S.* Albany strain 7205.00 (Table 1). A first set of historical MDR IncA/C plasmids isolated at the end of the 1960′s like the reference IncA/C plasmid pRA1, i.e. pIP40a (from *Pseudomonas aeruginosa* in 1969), pR16a (from *Providencia Stuartii* in 1966), and pR55 (from *Klebsiella pneumoniae* in 1969) that was firstly used to experimentally mobilize SGI1, was tested in SGI1 mobilization assays (Table 1) [15], [21], [23], [24]. *E. coli* SGI1 transconjugants were obtained in all mobilization assays (Table 2). Transconjugants showed the antibiotic resistance profile of the SGI1 variant horizontally transferred and susceptibility to specific antibiotic markers of the different helper IncA/C plasmids used in the donor strains. All these results were confirmed by PCRs on *E. coli* transconjugants (data not shown). Depending on the helper conjugative IncA/C plasmid used, and also on the *S. enterica* SGI1 donor strain, the SGI1 transfer frequencies were found to range from 10^−3^ to 10^−5^. These results indicated that several conjugative IncA/C plasmids were able to mobilize *in trans* SGI1 or variants of it. Thus, these results strengthen the hypothesis that there is a specific relation between the IncA/C plasmid family and the ability to mobilize SGI1.

To further confirm this hypothesis, other current MDR IncA/C plasmids encoding the extended spectrum cephalsporinase CMY-2 were also tested in SGI1 mobilization assays (Table 1). Since 2000, the emergence of extended-spectrum cephalosporin-resistant *S. enterica* carrying IncA/C plasmid-mediated CMY-2 AmpC β-lactamase has been reported in numerous countries around the world. Such CMY-2 IncA/C plasmids have been also identified in *E. coli* isolates [8], [9]. Two CMY-2 IncA/C plasmids p13688 and p13956 isolated from *E. coli* animal strains were introduced by conjugation from an *E. coli* transconjugant to *S.* Agona derivative strain 959SA97ΔS009::*kan* or *S.* Agona derivative strain 47SA97Δ*xis*::*kan* [9]. These mutant strains contain a kanamycin marker in SGI1 that was necessary for the selection of SGI1 in mating experiments according to the large antibiotic resistance profile of these plasmids (Table 1). The ORF S009 initially annotated in the SGI1 sequence revealed to be non-functional, thus the insertion of the kanamycin marker gene would not affect the transfer of SGI1 (B. Doublet, unpublished results). Strain 47SA97Δ*xis*::*kan* has been previously described and the deletion of the *xis* gene in SGI1-C did not abolish the SGI1 transfer but slightly affected its transfer frequency [15]. Like with the previously tested IncA/C plasmids, these 2 conjugative CMY-2 IncA/C plasmids allowed the mobilization of SGI1 at frequencies ranging from 10^−5^ to 10^−6^ (Table 2). Two other current CMY-2 IncA/C plasmids, pAM04528 and pN418, isolated from representative strains of clonal epidemic *S.* Heidelberg and *S.* Newport in the U.S. were also studied to mobilize SGI1 [7], [8]. Conjugation experiments from the original *S. enterica* host strains to *E. coli* recipient strain failed for the CMY-2 IncA/C plasmid pAM04528 and yielded many *E. coli* transconjugants for the CMY-2 IncA/C plasmid pN418. Then, the CMY-2 IncA/C plasmid pN418 was introduced by conjugation into SGI1-F-carrying *S.* Albany strain 7205.00 and plasmid pAM04528 was transferred by electroporation into the same SGI1 donor strain. For both plasmids no *E. coli* SGI1 transconjugants were obtained in 3 independent assays of SGI1 mobilization (Table 2). Control of conjugative transfer of helper plasmid was positive for pN418 and as suspected, negative for pAM04528. As expected, these results indicated that a non-self-transferable IncA/C plasmid, i.e. pAM04528 from *S.* Newport, was not able to mobilize *in trans* SGI1. However surprisingly, the CMY-2 MDR IncA/C plasmid pN418 from *S.* Heidelberg, which is self-transmissible, was also not able to mobilize SGI1. These two plasmids have been previously described in different studies and seemed to be very similar [7], [8]. Thus, these data suggested that there might exist genetic differences between these two CMY-2 MDR IncA/C plasmids and also between the 2 others CMY-2 MDR IncA/C plasmids isolated from *E. coli* strains of this study that could explain the efficiency or not to mobilize SGI1. Moreover, the difference in SGI1 transfer rates ranging from 10^−3^ to 10^−6^ could be another result of transfer function differences between IncA/C plasmids. These results are in accordance with other studies which showed that transfer rates for different IncA/C plasmids can vary by as much as 10^4^ fold [5], [7], [25].

**Figure 1:**
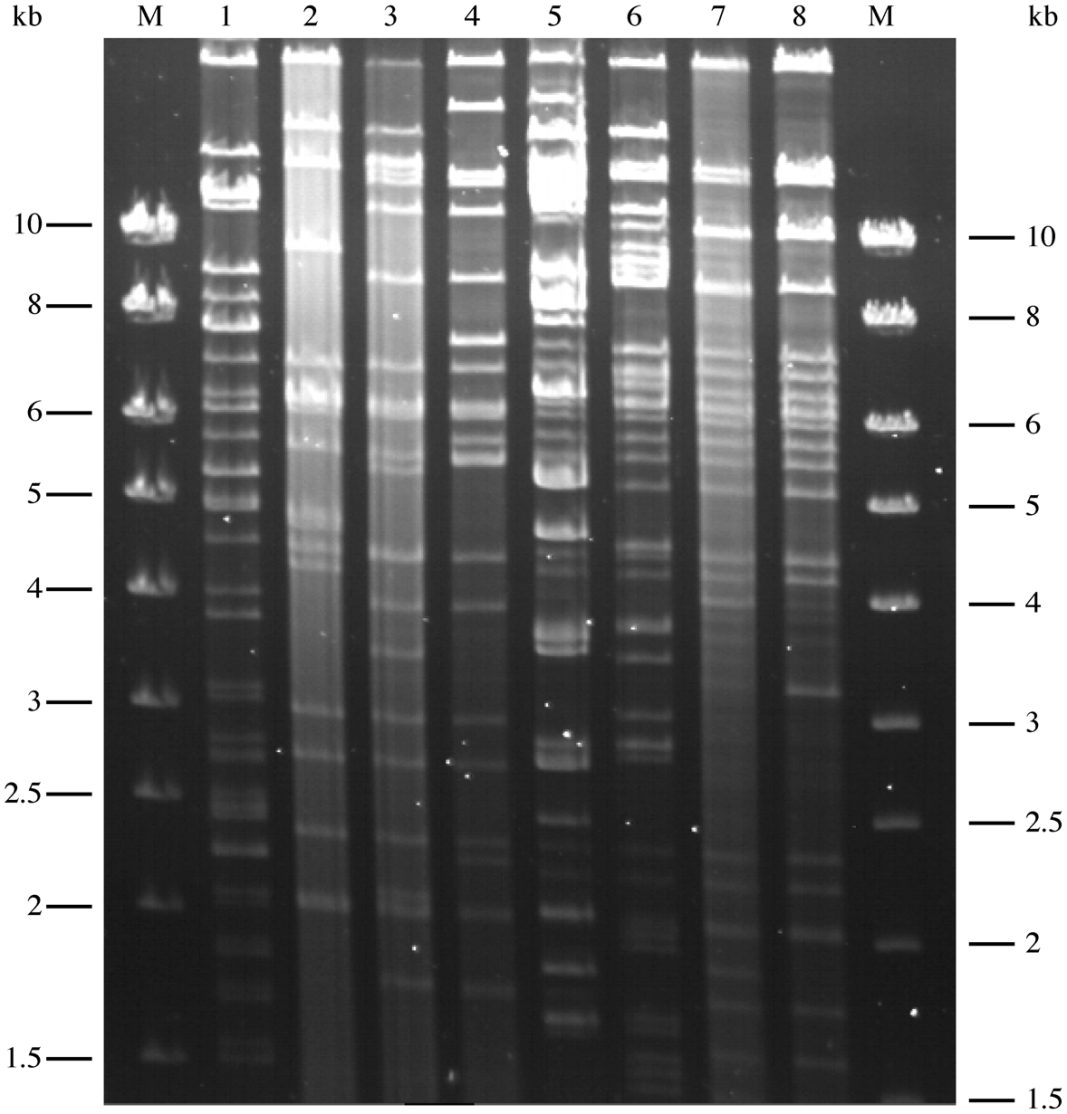
Restriction profile analysis of IncA/C plasmids digested by the restriction enzyme *Dra*I. Lane 1, pRA1; lane 2, pR55; lane 3, pR16a; lane 4, pIP40a; lane 5, p13956; lane 6, p13688; lane 7, pN418; lane 8, pAM04528; M, kbp molecular marker (Smartladder, Eurogentec, Seraing, Belgium).

**Figure 2:**
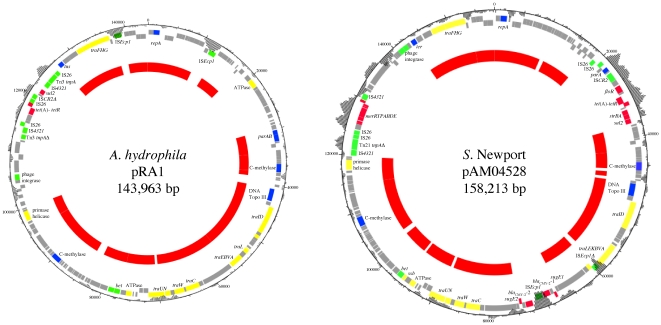
Circular representations of previously sequenced IncA/C plasmids pRA1 and pAM04528 and their common IncA/C backbone (red inner circle). Nucleotide composition (GC plot) is represented on each plasmid and genes were color coded, depending on functional annotations, as follows: plasmid replication/maintenance, blue; transposition/recombination, green; conjugative plasmid transfer, yellow; antimicrobial resistance, red; other functions/hypothetical proteins, gray. Distance scales in base pair are given around each map. Sequences and annotations of pRA1 and pAM04528 are accessible in GenBank database under accession numbers FJ705807 and FJ621587, respectively.

A comparative genetic analysis of the large IncA/C MDR plasmids used in this study was set up based on restriction profile and comparison of two sequenced plasmids pRA1 and pAM04528 to assess their genetic relationship and to determine if their transfer efficiency could be explained by particular traits. According to the full-length sequences of plasmids pRA1 and pAM04528, enzymatic digestion of the historical and current IncA/C MDR plasmids was performed using restriction enzyme *Dra*I. Among the IncA/C MDR plasmids studied, the historical plasmids pR55, pR16, and pIP40a showed related *Dra*I patterns being only different by few fragments of different sizes (Figure 1). The same result was also observed for the 4 current epidemic CMY-2 IncA/C MDR plasmids, i.e. p13956, p13688, pN418, and pAM04528 (Figure 1). However, this second set of plasmids presented really distinct profiles compared to the historical ones. Interestingly, the IncA/C reference plasmid pRA1 revealed a distinct *Dra*I profile from all other IncA/C plasmids (Figure 1). Sequence comparison of the two sequenced IncA/C plasmids pRA1 and pAM04528 used in this study allowed to define a common IncA/C plasmid backbone between these 2 plasmids which represents around 100 kb in size indicated as an inner red circle in Figure 2. This common IncA/C plasmid backbone showed an overall genomic synteny and a nucleotide identity ranging from 88 to 94%. Moreover, the functional predictions inferred from the plasmid annotations showed that this IncA/C backbone corresponds to essential functions of a plasmid lifestyle such as replication, maintenance, and conjugative transfer (Figure 2). The main regions of difference between these plasmids correspond to different antibiotic resistance gene clusters and insertion sequences as shown by the deviating nucleotide composition (Figure 2), which seem to be acquired by insertion/transposition events. It is worth to note that although a large common IncA/C backbone is shared, the *Dra*I restriction profiles of these plasmids are very different from each other (Figure 1).

A recent study from Lindsey et al. showed that the combination of *Salmonella*, IncA/C plasmids, and MDR is very ancient. In their work, they demonstrated an interesting relation between the presence/absence of IncA/C MDR plasmids, the presence/absence of SGI1 and epidemic successful serovars of *S. enterica* like Typhimurium DT104 and Newport [6]. MDR plasmids belonging to the IncA/C family are widely distributed among *Salmonella* and other Enterobacteriaceae from animal sources and have caused considerable concern in public health community [6], [7]. Moreover, a stronger association of MDR with IncA/C replicon was observed in *Salmonella* than with other replicon types [6]. This association is probably related to a highly conserved IncA/C plasmid backbone into which horizontally acquired antibiotic resistance fragments were integrated in few sites [25].

An important trait for the epidemic spread of IncA/C plasmids lies in their conjugative self-transmissibility. Welch et al. demonstrated that a large majority of IncA/C plasmids found in *Salmonella* strains were able to transfer horizontally [25]. However, several studies showed that the conjugal transfer could not be demonstrated for all CMY-2 MDR IncA/C plasmids of epidemic *S.* Newport isolates in the U.S. [25]. Poole et al. also observed that CMY-2 MDR IncA/C plasmids from *Salmonella* strains rarely transferred when it was the only replicon detected in the donor strain [5]. Regarding the majority of large MDR IncA/C plasmids sequenced and their self-transferability when it is known, there are few differences, which could explain the conjugative efficiency or not. The only difference identified involving *tra* genes was a partial duplication of *traC* genes associated with the duplication of the *bla*_CMY-2_ locus in non-self-transferable plasmid identified in *S*. Newport isolates [7], . Only one larger fragment upstream of the *parAB* cluster is absent from pAM04528 but present on the IncA/C reference plasmid pRA1 [7]. However, this region contains several ORFs of unknown function and only one putative ATPase which could be implicated in transfer function. When transferability of sequenced plasmids were known, no correlation was observed between the presence of this region and the self-transferability [5], [7], [8]. The loss of transfer efficiency of current epidemic CMY-2 IncA/C plasmids of *S*. Newport isolates may be due to numerous insertion sequences present on these plasmids compared to historical ones. However, several questions remain to be answered: (i) Why the self-transferable IncA/C plasmid pN418 is unable to mobilize SGI1; and (ii) What and/or where is the specific link between IncA/C transferability and SGI1 mobilization. To answer these questions, further studies need to be undertaken on specific transfer elements of SGI1 to connect them to IncA/C transfer functions.

In summary, we have shown that the MDR genomic island SGI1 is specifically mobilized *in trans* by the conjugative IncA/C plasmid family. Depending on the IncA/C plasmid used, the conjugative transfer of SGI1 occurred at frequencies ranging from 10^−3^ to 10^−6^ transconjugants per donor. Of particular concern, some large IncA/C MDR plasmids carrying the extended-spectrum cephalosporinase *bla*_CMY-2_ gene were shown to mobilize *in trans* SGI1. To the best of our knowledge, this study represents the first description of a specific relation between an incompatibility plasmid group and the specific mobilization of another mobile genetic element. Related IncA/C MDR plasmids have been detected in several fish pathogens such as *Aeromonas*, *Yersinia*, *Photobacterium*, *Edwardsiella* indicating that IncA/C plasmids mediates environmental MDR dissemination between bacteria from mammalian enteric flora and from aquatic ecosystem [7], [8], [25]–[27]. Thus, aquatic environment may be a favourable ecologic niche where horizontal transfer takes place between different bacterial genera. More than just their own dissemination, conjugative IncA/C plasmids may also contribute to the spread of the antibiotic resistance genomic island SGI1 and its close-derivatives among enteric pathogens and potentially more widely.

## Materials and Methods

### Bacterial strains, media, plasmids and antibiotic susceptibility testing

The *Salmonella* strains harbouring SGI1 or variants of it used in conjugation experiments are described in Table 1. *S. enterica* strains 959SA97 (serovar Agona harbouring SGI1), 47SA97 (serovar Agona harbouring the SGI1-C variant), 7205.00 (serovar Albany harbouring the SGI1-F variant) were used as donor strains [15]. *E. coli* K-12 strain BM14 was used as recipient strain. All strains were grown at 37°C in brain heart infusion broth or agar plates. The *Salmonella*-*Shigella* (SS) medium with addition of appropriate antibiotics was used for selection of *S. enterica* donors and *E. coli* SGI1 transconjugants in mobilization experiments. Conjugative helper plasmids of different host origins used in mobilization experiments are listed in Table 1. These plasmids (except pAMO4528 and pN418) have been previously described to be self-transmissible (Tra^+^) [see references in Table 1]. Donor, recipient, and transconjugant strains were screened for antibiotic resistance by the disk diffusion method on Mueller-Hinton agar plates [28]. Susceptibility was tested using disks containing the following antibiotics: Amx (10 µg), Chl (30 µg), Kan (30 IU), Gen (15 µg), Str (10 IU), Sul (200 µg), Tet (30 IU), Tmp (5 µg) and for third generation cephalosporins, ceftriaxone (30 µg), cefepime (30 µg), and ceftiofur (30 µg). All antibiotic disks were purchased from BioRad (Marnes la Coquette, France).

### Bacterial conjugations

Bacterial conjugation was performed to introduce the different helper plasmids into the SGI1-containing *S. enterica* strains. Briefly, end-log exponential phase liquid cultures of an *E. coli* donor containing helper plasmid and a recipient SGI1-carrying *S. enterica* strain were mixed together in a approximately 1∶4 ratio. After overnight incubation without shaking at 37°C, the mating was streaked on appropriate selective SS agar plates. Antibiotics for which resistances were conferred by SGI1, were used to select *Salmonella* SGI1 recipient strains and antibiotic resistances displayed by helper plasmids were chosen to select transconjugants.

SGI1 mobilization assays were performed by mixing *S. enterica* SGI1 donor strain harbouring different helper plasmid and the sodium azide-resistant *E. coli* recipient strain BM14 together with a donor-to-recipient ratio of 4∶1. This broth was incubated overnight at 37°C without shaking. The next day, the cells were streaked on appropriate selective SS agar plates. Sodium azide (500 µg/ml) was used to select against *Salmonella* donor cells, and Str (50 µg/ml) and/or Tet (10 µg/ml) and/or Tmp (40 µg/ml) to select against unmated recipient cells. *Salmonella* donors in the mating were numbered on SS agar plates supplemented with antibiotics selecting for SGI1 (Str, Tet, Tmp) and the helper plasmid (antibiotic resistance only conferred by the plasmid and not by SGI1). Appropriate control plates were performed for the conjugative transfer of the helper plasmid in each mating experiment. The SGI1 frequency of transfer was determined by dividing the number of *E. coli* SGI1 transconjugants by the number of *Salmonella* donor cells.

### SGI1 PCR detection, PCR-based replicon typing, IncA/C plasmid restriction profile, and sequence analysis

Detection of SGI1 and its location in *Salmonella* strains and in *E. coli* transconjugants were performed using primers corresponding to the left and right junctions in the chromosome as described previously [15], [23]. Primers U7-L12 (located in the *trmE* gene) and 104D (located in the *yidY* gene) corresponding to the *Salmonella* chromosome and primers EcU7-L12 and Ec104D (located in the *tnaL* gene) corresponding to the *E. coli* chromosome were used to assess SGI1 junctions in its specific attachment site in *Salmonella* and *E. coli*, respectively (Table S1).

Conjugative helper plasmids in *E. coli* donor strains were typed by the PCR-based replicon typing as previously described [3]. The same method was applied to confirm the presence of the different helper plasmids in the *Salmonella* SGI1 donor strains. Additional PCRs were performed to amplify known resistance genes carried by the helper plasmids especially for ESC resistance genes *bla*_TEM_, *bla*_CTX-M_, *bla*_CMY_, and for phenicol resistance genes, *cat* and *floR* (Table S1).

To assess the genetic relationship between IncA/C helper plasmids used to mobilize SGI1, plasmid DNA was extracted and purified from *E. coli* host strains with the QIAGEN plasmid midi kit (Courtaboeuf, France) used according to the manufacturer's recommendations. According to the sequence of the IncA/C reference plasmid pRA1, plasmid DNA was digested with the restriction enzyme *Dra*I (Promega, Charbonnières-les-Bains, France). Fragments of DNA were separated by electrophoresis in 0.6% ultra pure DNA grade Agarose gel (BioRad, Marnes la Coquette, France).

Sequence comparisons at the nucleotide level were carried out with the BLAST algorithm using the Artemis Comparison Tool [29]. Plasmid maps of pRA1 and pAM04528 were designed using DNA plotter according the annotations in GenBank database under accession numbers FJ705807 and FJ621587, respectively. Their common IncA/C backbone was assigned for region of high nucleotide identity and of genomic synteny.

## Supporting Information

Table S1 **SGI1 mobilization assays by different incompatibility group plasmids.**
(DOC)
