# Supplementary material for: The Salmonella Genomic Island 1 Is Specifically Mobilized In Trans by the IncA/C Multidrug Resistance Plasmid Family
Source: PLoS One. 2010 Dec 20;5(12):e15302. doi: 10.1371/journal.pone.0015302 (PMC3004903; doi:10.1371/journal.pone.0015302)
Supplement: Table S1 — SGI1 mobilization assays by different incompatibility group plasmids. (DOC) [file pone.0015302.s001.doc]

**Table S1. SGI1 mobilization assays by different incompatibility group plasmids.**

| *S. enterica* donor strain | | SGI1 variant | Conjugative plasmid | SGI1 transfer frequency^a^ |
| --- | --- | --- | --- | --- |
| **Field strain** | |  |  |  |
|  | Agona 777SA01 | SGI1-A | IncI1 p777-SA-01 (TEM-52) | < 10^-9^ |
|  | Typhimurium 04-3486 | SGI1 | IncI1 p04-3486 (TEM-52) | < 10^-9^ |
| Transconjugant strain | | |  |  |
|  | Agona 959SA97 | SGI1 | IncFI pOX38 | < 10^-9^ |
|  | Agona 959SA97 | SGI1 | IncFII R1-16 | < 10^-9^ |
|  | Agona 959SA97 | SGI1 | IncHI2 pCEB6542 | < 10^-9^ |
|  | Agona 959SA97 | SGI1 | IncHI2 p1639-SA-00 | < 10^-9^ |
|  | Agona 959SA97 | SGI1 | IncHI2 p142-SA-01 | < 10^-9^ |
|  | Agona 959SA97 | SGI1 | IncHI2 p3464b | < 10^-9^ |
|  | Agona 959SA97 | SGI1 | IncI1 p777-SA-01 | < 10^-9^ |
|  | Agona 959SA97 | SGI1 | IncI1 p04-3486 | < 10^-9^ |
|  | Agona 959SA97 | SGI1 | IncI1 R112 | < 10^-9^ |
|  | Agona 959SA97 | SGI1 | IncL/M R69 | < 10^-9^ |
|  | Agona 959SA97 | SGI1 | IncN RPC3 | < 10^-9^ |
|  | Agona 959SA97 | SGI1 | IncP RP4 | < 10^-9^ |
|  | Agona 959SA97 | SGI1 | IncW RSa | < 10^-9^ |

^a^ the frequency of transfer was calculated by dividing the number of SGI1 transconjugants by the number of SGI1 donor cells. Transfer frequencies correspond to the result of one experiment that has been repeated two times and showing the same results.
